# A computer program for intra-laboratory quality control

**DOI:** 10.1155/S1463924684000298

**Published:** 1984

**Authors:** Cecilia Zuppi, Giuliano Barbaresi, Maria Luisa Gozzo, Bruno Zappacosta

**Affiliations:** Istituto di Chimica Biologica Laboratorio di Chimica Clinica, Policlinico Universitario A. Gemelli Università Cattolica del Sacro Cuore Largo A. Gemelli 8 Roma 00168 Italy


Journal of Automatic Chemistry, Volume 6, Number 3 (July–September 1984), pages 155–157

### A computer program for intra-laboratory quality control

**Cecilia Zuppi, Giuliano Barbaresi, Maria Luisa Gozzo and Bruno Zappacosta**

*Istituto di Chimica Biologica, Laboratorio di Chimica Clinica, Policlinico Universitario A. Gemelli, Università Cattolica del Sacro Cuore, Largo A. Gemelli 8, 00168 Roma, Italy*

### Introduction

Intra-laboratory quality-control (intra-lab QC) is the best way of checking the performance of single components of analytical systems (methods, instruments, technicians) [1]. However, the time-consuming and tedious statistical methods necessary, if manually performed, provide late information that reduces the validity of intra-lab QC.

In order to make this type of quality control more useful, a minicomputer program, which automates mathematical and graphical procedures, has been developed. The program assures rapid, unique and unequivocal interpretation of results by using a reliability index (RI) [2 and 3].

### Materials and methods

### *Hardware*

A P6060 minicomputer (Ing. C. Olivetti & C., S.p.A., Ivrea, Italy) was used with a 32 Kbyte ROM and a 400 Kbyte RAM on floppy disks.

### *Software*

The program was written in BASIC; a complete listing of the program is available from the authors.

### *Control sera*

Commercially available control sera with two known levels of analyte concentration were used. The control sera are alternatively analysed every 15 to 20 samples.

### *Statistical methods*

The reliability index and RI cusum ($Q$) [4] are used to evaluate analytical results in real time; the RI is derived from Whitehead's formulae for precision (PI) and accuracy (AI) indexes [5]. These indexes express, in terms of standard deviation, the differences between the previously observed and the obtained value (PI) and between the observed value and the expected one (AI). In order to obtain an index representative of both precision and accuracy the following formula was used:

$$\mathrm{RI} = \frac{(X_a - \bar{x}_c)}{\mathrm{SD}_c} . \ 100$$

where $x_a$ is the found value, $\bar{x}_c$ is the cumulative mean, and $\mathrm{SD}_c$ is the cumulative standard deviation. The cumulative mean is considered to be the best estimate of a quantity for a particular material using a defined analytical method, and the cumulative standard deviation the best expression of method variability [6]. Statistically, RI is equivalent to the Z-score or to the standard deviation interval (SDI) multiplied by 100. Cusum is the cumulative sum of RIs. Its value progressively increases if there is no variation in the RI sign; it begins again from zero if a sign variation appears.

The statistical parameters are calculated monthly to allow retrospective quality-control. Monthly means and standard deviations on untruncated, truncated, and the cumulated results of two control materials are calculated. Truncated data means and related standard deviations are obtained after an iterative truncation deletes outliers (±3 SD). The truncated data are summed with those from the previous months and are used to calculate cumulative means and standard deviations. Control charts [7], and two sample plots [8] are printed by means of the P6060's printer.

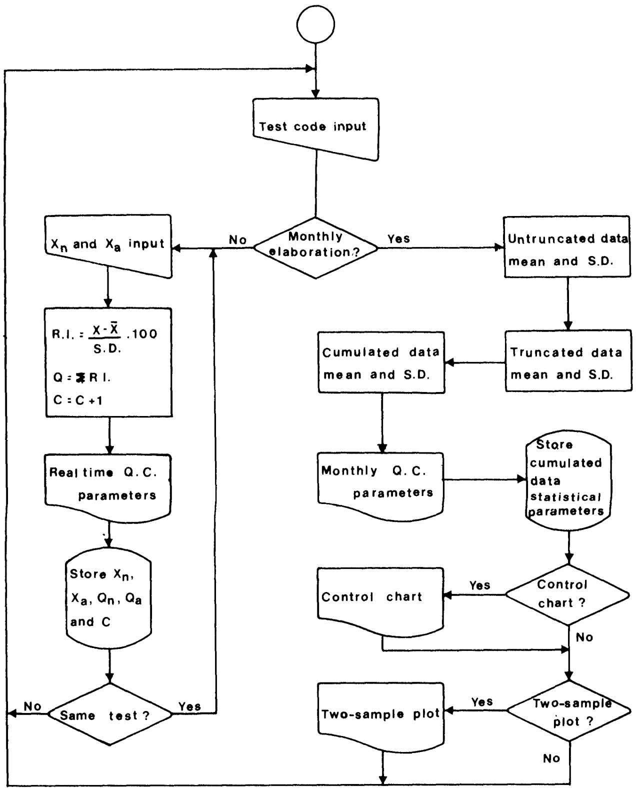

*Figure 1. Flowchart.*

### *System description*

The program manages two direct-access external files: the first is reserved for numerical and alphanumerical parameters, the

GAMMA-GT: REAL TIME ELABORATION (5 NOVEMBER 1982)

| | | | | | |
|---|---|---|---|---|---|
| 6) | NORMAL LEVEL: | U.I./L | ●63·0 ▽(64·8) | R.I.= −92 | Cusum= −92 |
| 6) | ELEVATED LEVEL: | U.I./L | 188·0 (187·1) | R.I.=27 | Cusum=27 |
| 7) | NORMAL LEVEL: | U.I./L | 67·0 (64·8) | R.I.=108 | Cusum=108 |
| 7) | ELEVATED LEVEL: | U.I./L | 192·0 (187·1) | R.I.=141 | Cusum=168 |

CPK: REAL TIME ELABORATION (5 NOVEMBER 1982)

| | | | | | |
|---|---|---|---|---|---|
| 5) | NORMAL LEVEL: | U.I./L | 152·0 (154·4) | R.I.= −55 | Cusum= −55 |
| 5) | ELEVATED LEVEL: | U.I./L | 296·0 (299·7) | R.I.= −83 | Cusum= −83 |
| 6) | NORMAL LEVEL: | U.I./L | 151·0 (154·4) | R.I.= −77 | Cusum= −132 |
| 6) | ELEVATED LEVEL: | U.I./L | 302·0 (299·7) | R.I.=51 | Cusum=51 |

*Figure 2. Real time elaboration. ● Found value. ▽ Expected value.*

MONTHLY ELABORATION: NOVEMBER 1982

GAMMA-GT (NORMAL LEVEL): RESULTS AND RELATED RELIABILITY INDEXES

| | | | | | | | | | |
|---|---|---|---|---|---|---|---|---|---|
| 1) | 65·0 (8) | 2) | 65·0 (8) | 3) | 62·0 (−143) | 4) | 64·0 (−42) | | |
| 5) | 66·0 (58) | 6) | | 7) | 67·0 (108) | 8) | 63·0 (−92) | | |
| 9) | 64·0 (−42) | 10) | 63·0 (−92) | 11) | 67·0 (108) | 12) | 67·0 (108) | | |
| 13) | 63·0 (−92) | 14) | 65·0 (8) | 15) | 67·0 (108) | 16) | 62·0 (−143) | | |
| 17) | 64·0 (−42) | 18) | 73·0 (409) | 19) | 65·0 (8) | 20) | 71·0 (309) | | |
| 21) | 65·0 (8) | 22) | 65·0 (8) | 23) | 64·0 (−42) | 24) | 61·0 (−193) | | |
| 25) | 63·0 (−92) | 26) | 65·0 (8) | 27) | 64·0 (−42) | 28) | 64·0 (−42) | | |
| 29) | 65·0 (8) | 30) | 66·0 (58) | 31) | 65·0 (8) | | | | |

GAMMA-GT (NORMAL): N.=31 (U.D.) MEAN=64·9 SD=2·43 CV%=3·7

GAMMA-GT (ELEVATED LEVEL): RESULTS AND RELATED RELIABILITY INDEXES

| | | | | | | | |
|---|---|---|---|---|---|---|---|
| 1) | 188·0 (27) | 2) | 186·0 (−31) | 3) | 183·0 (−117) | 4) | 186·0 (−31) |
| 5) | 187·0 (−2) | 6) | 188·0 (27) | 7) | 192·0 (141) | 8) | 191·0 (113) |
| 9) | 185·0 (−59) | 10) | 189·0 (55) | 11) | 192·0 (141) | 12) | 190·0 (84) |
| 13) | 188·0 (27) | 14) | 186·0 (−31) | 15) | 188·0 (27) | 16) | 186·0 (−31) |
| 17) | 180·0 (−203) | 18) | 196·0 (256) | 19) | 185·0 (−59) | 20) | 188·0 (27) |
| 21) | 182·0 (−146) | 22) | 187·0 (−2) | 23) | 190·0 (84) | 24) | 184·0 (−88) |
| 25) | 186·0 (−31) | 26) | 188·0 (27) | 27) | 180·0 (−203) | 28) | 186·0 (−31) |
| 29) | 186·0 (−31) | 30) | 189·0 (55) | 31) | 190·0 (84) | | |

GAMMA-GT (ELEVATED):N.=31 (U.D.) MEAN=187·1 SD=3·36 CV%=1·7

GAMMA-GT (NORMAL):N.=29 (T.D.) MEAN= 64·4 SD=1·57 CV%=2·4

GAMMA-GT (ELEVATED):N.=31 (T.D.) MEAN=187·1 SD=3·36 CV%=1·7

GAMMA-GT (NORMAL):N.=231 (C.D.) MEAN=64·8 SD=1·95 CV%=3·0
GAMMA-GT (ELEVATED):N.=242 (C.D.) MEAN=187·1 SD=3·46 CV%=1·9

*Figure 3. Monthly elaboration. U.D.=Untruncated data; T.D.=truncated data; C.D.=cumulated data.*

second for memorization of the analytical results obtained from controls, two by two. The first file, 10 000 bytes, is made up by 125 records of 20 words (4 bytes for each one). Six words from each record are reserved for the test description (for example Gamma-GT IU/L); the numbers of cumulated data ($N_n$ and $N_a$), the averages ($X_n$ and $X_a$), and the standard deviations ($SD_n$ and $SD_a$) of normal ($n$) and abnormal ($a$) sera, respectively, are stored using the next six words. The last eight words are reserved for data summation ($\Sigma Xi_n$ and $\Sigma Xi_a$) and for summation of the square data ($\Sigma Xi_n^2$ and $\Sigma Xi_a^2$) in double precision [9].

The initial mean and standard deviation are obtained after a testing period under optimal analytical conditions.

The second file (201 500 bytes) is made up by 125 records of 403 words. The first three words of each record are reserved for the memorization of the number of introduced pairs (C) and of the cusum current value ($Q_n$ and $Q_a$). The following words are reserved for sequentially storing the result pairs ($Xi_n$ and $Xi_a$). The key for direct access to external files is obtained either by using the test code or a combination of this code with the value of the data counter.

These files permit storage and analysis of the results of intra-lab QC obtained from 125 analytes. A fixed monthly limit of 200 results for each analyte at two concentration levels is imposed. However, the number of controlled analytes can be changed and a proportional space for it can be reserved; in this case a conversion table, stored in an internal file, permits direct access to the external files.

In everyday use, the program requests the operator to input a test code and result pairs at two concentration levels (figure 1). An optional input validation routine is also included. RI and cusum value for each concentration level are immediately calculated and printed (figure 2). Analytical result pairs, monthly value of the test counter and cusum values are stored. The operator can request the program to provide a monthly elaboration (figure 3), to print out control charts (figure 4) and two sample plots (figure 5).

### Discussion

Reliability of laboratory results assures their clinical usefulness. In the last 10 years there has been a progressive improvement in the analytical methods and technology available for quality control. Many inter-lab QC programs have been suggested and developed, but their success might be due to their easy execution

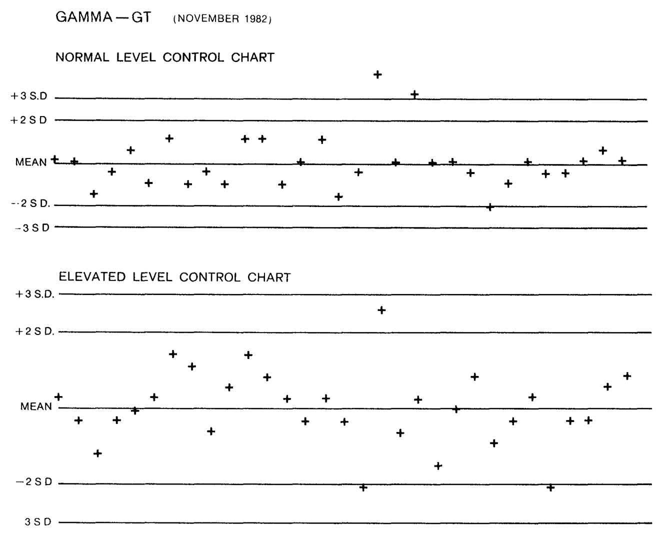

*Figure 4. Monthly control charts.*

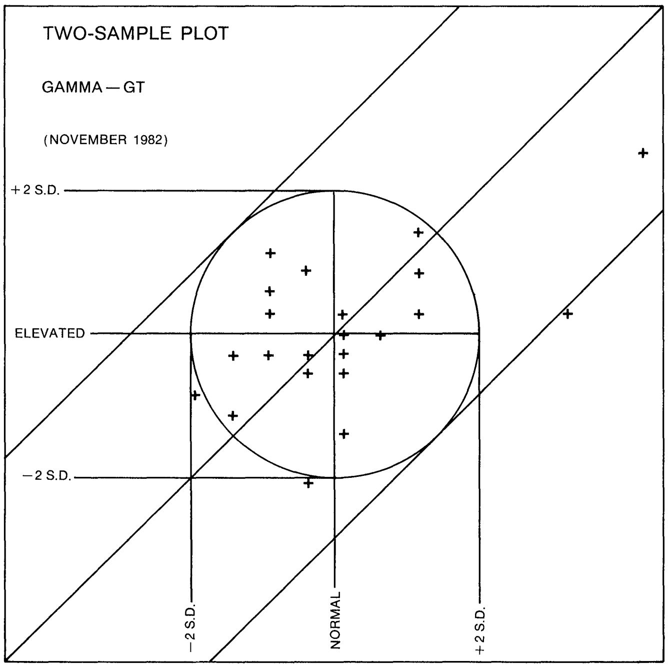

*Figure 5. Two-sample plot.*

rather than their effectiveness as a routine method. An inter-lab control program assures accuracy in relation to other laboratories, but these programs are generally sporadic, restricted in terms of test numbers, and always untimely; so they cannot assure world-wide stability of analytical systems.

Intra-lab QC programs, on the other hand, require manual development of mathematical and graphical procedures and are tedious and time-consuming, also these programs do not allow real time control. So manual intra-lab QC is limited to simple visual judging of control sera results, while the elaboration of data is reserved to qualified personnel only.

In order to improve intra-lab QC, the usual mathematical and graphical elaborations have been automated and the statistical procedures improved with an efficient parameter: RI. This unitless index assures a unique and unequivocal interpretation of control sera results and provides for immediate error detection.

Moreover, simultaneous calculation of the RI cusum permits a quick evaluation of possible systematic errors. Retrospective judgement of analytical effectiveness is obtained from the development of usual statistical parameters on untruncated, truncated, and cumulated data. Monthly means and SDs of untruncated data describe inaccuracy and imprecision, respectively, while parameters of truncated data are cumulated and used for calculated of RIs, assuring sensitivity to the real time control procedures. Finally, outliers frequency is a further parameter for evaluating analytical performance. Control charts and two sample plots show graphically the analytical variability, magnitude, and kind of systematic error. Now that all memorization and calculation procedures are perfomed by minicomputer, the management and interpretation problems of intra-lab QC have been overcome in the Clinical Chemistry Laboratory at the Università Cattolica del Sacro Cuore. The program has been well accepted, even by personnel who had no previous computing and statistical experience.

### References

1. Groth, T., *Scandinavian Journal of Clinical and Laboratory Investigation*, **40** (1980), 47.
2. Giocoli, G., Gozzo, M. L., Barbaresi, G. and Miggiano, G., *Giornale Italiano di Chimica Clinica*, **6** (1981), 4.
3. Barbaresi, G., Martorana, G. E., Zuppi, C. and Castelli, A., *Computers in Biology and Medicine*, **13** (1983), 7.
4. Rowlands, R. J., Wilson, D. W., Nix, A. B. J., Kemp, K. W. and Griffiths, K., *Clinica Chimica Acta*, **108** (1980), 393.
5. Whitehead, T. P., *Advances in Clinical Chemistry*, **19** (1977), 175.
6. Burnett, R. W., *Clinical Chemistry*, **21** (1975), 1935.
7. Levey, S., and Jenning, E. R., *American Journal of Clinical Pathology*, **20**, (1950), 1059.
8. Skendzel, L. P. and Youden, W. J., *American Journal of Clinical Pathology*, **51** (1969), 161.

### COLLOQUIUM SPECTROSCOPIUM INTERNATIONALE XXIV

*To be held from 15 to 21 September 1985 in Garmisch-Partenkirchen, FR Germany*

CSI XXIV will touch upon all areas of analytical chemistry; an international group of spectroscopists is expected to attend. Programme topics include:

*Basic theory and methods—*

- Atomic emission spectroscopy
- Atomic absorption spectrometry
- Atomic fluorescence spectrometry
- Infra-red and Raman spectroscopy
- Molecular spectroscopy (UV and Vis)
- Laser spectroscopy
- Mass spectroscopy (organic and inorganic)
- Standard reference materials etc.

*Analytical applications for specific problems—*

- Analysis of metals
- Analysis of different industrial products
- Geochemical analysis
- Biological, clinical and pharmaceutical analysis
- Analysis in agriculture and nutrition chemistry
- Environmental analysis.

*The second circular will soon be available from CSI XXIV, Organisationsbüro, Institut für Spektrochemie und angewandte Spektroskopie, Postfach 778, D 4600 Dortmund 1, FR Germany.*

